# A second monoclinic polymorph of 2-(3,5-dimethyl-1*H*-pyrazol-1-yl)-2-hy­droxy­imino-*N*′-[1-(pyridin-2-yl)ethyl­idene]acetohydrazide

**DOI:** 10.1107/S1600536813009628

**Published:** 2013-04-20

**Authors:** Maxym O. Plutenko, Rostislav D. Lampeka, Matti Haukka, Ebbe Nordlander

**Affiliations:** a Department of Chemistry, National Taras Shevchenko University, Volodymyrska Street 64, 01601 Kyiv, Ukraine; b Department of Chemistry, University of Jyvaskyla, PO Box 35, FI-40014 Jyvaskyla, Finland; c Inorganic Chemistry, Center for Chemistry and Chemical Engineering, Lund University, Box 124, SE-221 00 Lund, Sweden

## Abstract

The title compound, C_14_H_16_N_6_O_2_, is a second monoclinic polymorph of 2-[1-(3,5-dimeth­yl)pyrazol­yl]-2-hy­droxy­imino-*N*′-[1-(2-pyrid­yl)ethyl­idene] acetohydrazide, with two crystallographically independent mol­ecules per asymmetric unit. The non-planar mol­ecules are chemically equal having similar geometric parameters. The previously reported polymorph [Plutenko *et al.* (2012 ▶). *Acta Cryst.* E**68**, o3281] was described in space group *Cc* (*Z* = 4). The oxime group and the O atom of the amide group are *anti* with respect to the C—C bond. In the crystal, mol­ecules are connected by N—H⋯N hydrogen bonds into zigzag chains extending along the *b* axis.

## Related literature

For uses of oxime ligands, see: Penkova *et al.* (2009 ▶); Kanderal *et al.* (2005 ▶). For uses of oximes having additional donor functions as versatile ligands, see: Fritsky *et al.* (1998 ▶, 2004 ▶, 2006 ▶), Kanderal *et al.* (2005 ▶), Onindo *et al.* (1995 ▶); Sliva *et al.* (1997 ▶). For related structures, see: Duda *et al.* (1997 ▶); Kanderal *et al.* (2005 ▶); Krämer & Fritsky (2000 ▶); Moroz *et al.* (2010 ▶, 2012 ▶); Sliva *et al.* (1997 ▶); Świątek-Kozłowska *et al.* (2000 ▶); Mokhir *et al.* (2002 ▶); Penkova *et al.* (2010 ▶); Strotmeyer *et al.* (2003 ▶); Fritsky *et al.* (2000 ▶). For structure of the first polymorph, see Plutenko *et al.* (2012 ▶). For the synthesis, see: Kozikowski & Adamczyk (1983 ▶).
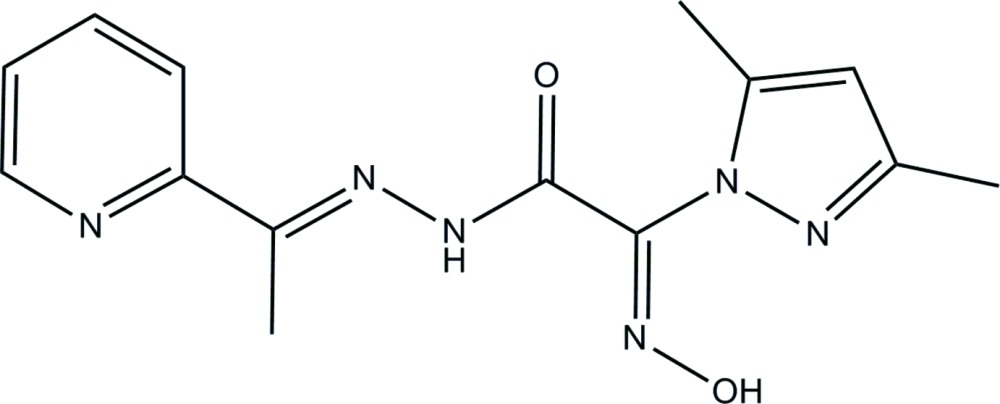


## Experimental

### 

#### Crystal data

C_14_H_16_N_6_O_2_
*M* *_r_* = 300.33
Monoclinic, 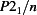

*a* = 19.4734 (4) Å
*b* = 7.7679 (2) Å
*c* = 19.8042 (4) Å
β = 97.552 (1)°
*V* = 2969.74 (11) Å^3^
*Z* = 8
Mo *K*α radiation
μ = 0.10 mm^−1^
*T* = 100 K
0.39 × 0.33 × 0.28 mm

#### Data collection

Bruker Kappa APEXII DUO CCD diffractometer
Absorption correction: multi-scan (*SADABS*; Sheldrick, 2008 ▶) *T*_min_ = 0.964, *T*_max_ = 0.974
64574 measured reflections
9973 independent reflections
8213 reflections with *I* > 2σ(*I*)
*R*_int_ = 0.027

#### Refinement

*R*[*F* ^2^ > 2σ(*F* ^2^)] = 0.038
*wR*(*F* ^2^) = 0.106
*S* = 1.03
9973 reflections
419 parameters
H atoms treated by a mixture of independent and constrained refinement
Δρ_max_ = 0.49 e Å^−3^
Δρ_min_ = −0.25 e Å^−3^

### 

Data collection: *APEX2* (Bruker, 2010 ▶); cell refinement: *SAINT* (Bruker, 2009 ▶); data reduction: *SAINT*; program(s) used to solve structure: *SHELXS97* (Sheldrick, 2008 ▶); program(s) used to refine structure: *SHELXL97* (Sheldrick, 2008 ▶); molecular graphics: *DIAMOND* (Brandenburg, 2008 ▶); software used to prepare material for publication: *SHELXL97*.

## Supplementary Material

Crystal structure: contains datablock(s) I, global. DOI: 10.1107/S1600536813009628/fk2070sup1.cif

Structure factors: contains datablock(s) I. DOI: 10.1107/S1600536813009628/fk2070Isup2.hkl

Supplementary material file. DOI: 10.1107/S1600536813009628/fk2070Isup3.mol

Supplementary material file. DOI: 10.1107/S1600536813009628/fk2070Isup4.cml

Additional supplementary materials:  crystallographic information; 3D view; checkCIF report

## Figures and Tables

**Table 1:** Hydrogen-bond geometry (Å, °)

*D*—H⋯*A*	*D*—H	H⋯*A*	*D*⋯*A*	*D*—H⋯*A*
O2—H2*O*⋯N5^i^	0.964 (17)	1.664 (17)	2.6193 (10)	170.1 (16)
O4—H4*O*⋯N10^ii^	0.978 (18)	1.670 (18)	2.6341 (10)	167.8 (17)

Symmetry codes: (i) 
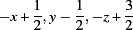
; (ii) 
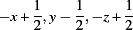
.

**Fig. 1.**
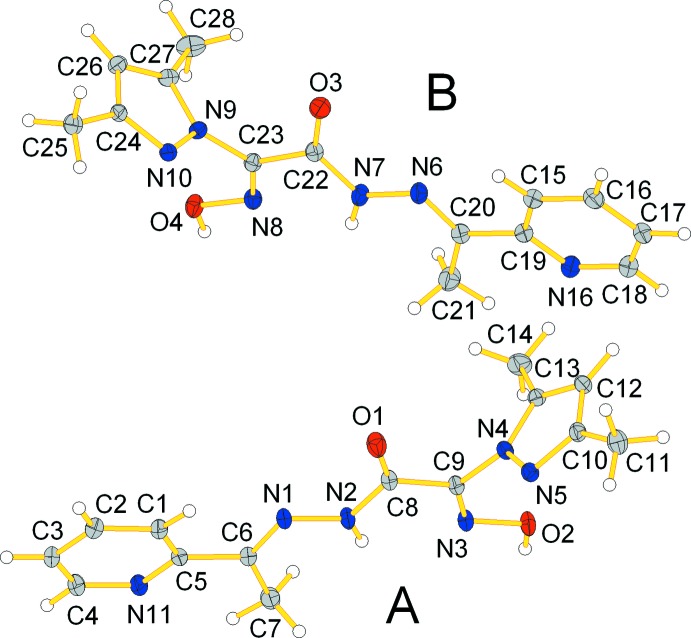
Molecular structure of molecules A and B of the title compound, with displacement ellipsoids shown at the 50% probability level. H atoms are drawn as spheres of arbitrary radii.

**Fig. 2.**
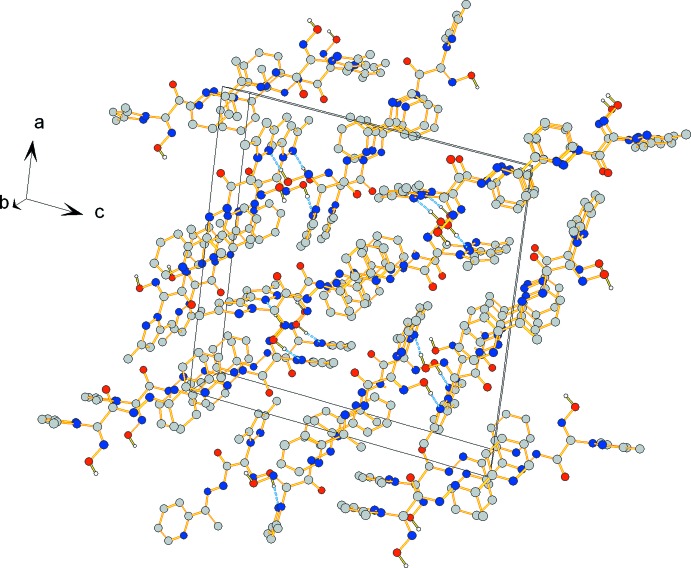
Crystal packing viewed along b-axis. Hydrogen bonds are indicated by dashed lines. H atoms not involved in H-bonds are omitted for clarity.

## supplementary crystallographic information

### Comment

Oximes are one of the most efficient bridging ligands class. Polydentate ligands containing both oxime and other donor functions are of special interest due to their potential for the bridging coordination modes and mediation of strong magnetic superexchange between metal ions (Penkova *et al.*, 2009; Kanderal *et al.*, 2005; Moroz *et al.*, 2010). Oxime ligands having the pyridyl groups in the molecule have been used in the preparation of complexes with a variety of transition metals, binding to metals in different modes most commonly as chelates or serving as bridge to metals, and the resulting species have been employed in molecular magnetism and supramolecular chemistry (Moroz *et al.*, 2010, 2012). Herein we report a second polymorph of 2-[1-(3,5-dimethyl)pyrazolyl]-2-hydroxyimino-N'-[1-(2-pyridyl)ethylidene] acetohydrazide (II) (Fig. 1). In comparison, the first polymorph I described previously (Plutenko *et al.*, 2012), crystallized in monoclinic space group Cc, Z = 4, while the title compound II crystallized in space group P21/n with Z = 8 (Fig. 1).

Bond lengths N-N', N-C and C-O of the amide group are 1.3693 (10), 1.3589 (11) and 1.2162 (11) respectively for molecule A and 1.3705 (11), 1.3603 (11) and 1.2156 (11) respectively for molecule B. Such bond lengths are typical for the protonated amide groups (Kanderal *et al.*, 2005). The oxime group is situated in *anti*- position to the amide group which was shown earlier in the structures of the amide derivatives of 2-hydroxyiminopropanoic acid (Onindo *et al.*, 1995; Sliva *et al.*, 1997; Fritsky *et al.*, 2006). The NC(=NOH)C(O)NH fragment deviates from planarity because of a twist between the oxime and the amide groups about the C(8)-C9 and C(22)-C(23) bonds; the O(1)-C(8)-C(9)-N(3) and O(22)-C(23)-C(9)-N(8) torsion angles are -175.953 (2)° and 164.073 (2)°. The bond lengths N-O and C-N of the oxime group are 1.3615 (9) and 1.2836 (11) Å respectively for A and 1.3645 (10) and 1.2819 (11) Å respectively for B. The bond lengths and angles within the oxime groups are normal and comparable to those in the related structures (Świątek-Kozłowska *et al.*, 2000; Mokhir *et al.*, 2002; Fritsky *et al.*, 1998). The C=N and N—O bond lengths in the oxime moiety of the molecule clearly indicates that the oxime group exists in the nitroso rather than in the isonitroso form (Duda *et al.*, 1997; Kanderal *et al.*, 2005; Fritsky *et al.*, 2004).

The C-C, C-N and N-N' (1,331 (0) – 1,409 (0) Å) bond lengths in the pyrazole ring exhibit normal values (Penkova *et al.*, 2010). The angles C-C'-C'', C-N-C', N-C-C' and N-N'-C are near 108°. The pyrazole ring deviates from the plane formed by other atoms of ligand molecule. The N(4)-C(9)-N(5)-N(6) and torsion angles are -103.589 (2) and 105.359 (2)° respectively. The C-N and C-C bond lengths in the pyridine rings are normal for 2-substituted pyridine derivatives (Fritsky *et al.*, 2000; Krämer *et al.*, 2000; Strotmeyer *et al.*, 2003).

In the crystal packing both molecules A and B are each connected by N-H···N hydrogen bonds, where the oxime nitrogen acts as donor and the pyrazole nitrogen atom acts as acceptor (Table 1). Thus, zig-zag supramolecular chains along b-axis are formed.

### Experimental

The present compound was synthesized according to Plutenko *et al.* (2012):

Synthesis of ethyl 2-[1-(3,5-dimethyl)pyrazolyl]-2-hydroxyiminoacetate: A mixture of ethyl 2-chloro-2-hydroxyiminoacetate synthesized according to Kozikowski *et al.* (1983) (0.906g, 6 mmol) and 3,5-dimethylpyrazol (1.152g, 12 mmol) in 10 ml of chloroform was left for evaporation in the air overnight. The resulting precipitate was crystallized from water. Yield: 1.12g (88 %).

Synthesis of 2-[1-(3,5-dimethyl)pyrazolyl]-2-hydroxyiminoacetohydrazide: A solution of hydrazine hydrate (0.57 ml, 60%, 10.6 mmol) in water was added to a solution of ethyl 2-[1-(3,5-dimethyl)pyrazolyl]-2-hydroxyiminoacetate (1.12g, 5.3 mmol) in methanol (30 ml). The resulting mixture was heating under reflux for 1.5 hours. After that solvent was evaporated and product was crystallized from methanol. Yield 0.5g (48 %).

Synthesis of 2-[1-(3,5-dimethyl)pyrazolyl]-2-hydroxyimino-N'-[1-(2-pyridyl)ethylidene]acetohydrazide: A solution of 2-[1-(3,5-dimethyl)pyrazolyl]-2-hydroxyiminoacetohydrazide (0.5g, 2.54 mmol) in methanol (30 ml) was treated with 2-acetylpyridine (0.307g, 2.54 mmol) and the mixture was heated under reflux for 3 hours. After that the solvent was evaporated in vacuum and the product was crystallized from methanol. Yield 0.65g (85 %).

### Refinement

OH, NH and CH_3_ hydrogen atoms were located from difference Fourier maps, other hydrogen atoms were positioned geometrically and all but H(N) and H(O) were refined at idealized positions riding on the parent atoms, with C—H = 0.95–0.98 Å, and *U*_iso_ = 1.2–1.5 *U*_eq_(parent atom). H(N) and H(O) atoms were refined freely with Uiso(H) = 1.2U_eq_(N) or 1.5U_eq_(O). All CH_3_ hydrogen atoms were allowed to rotate but not to tip. The highest peak is located 0.74 Å from atom C9 and the deepest hole is located 1.03 Å from atom N4.

### Crystal data

**Table:**

C_14_H_16_N_6_O_2_	*F*(000) = 1264
*M**_r_* = 300.33	*D*_x_ = 1.343 Mg m^−^^3^
Monoclinic, *P*2_1_/*n*	Mo *K*α radiation, λ = 0.71073 Å
Hall symbol: -P 2yn	Cell parameters from 9866 reflections
*a* = 19.4734 (4) Å	θ = 2.8–31.6°
*b* = 7.7679 (2) Å	µ = 0.10 mm^−^^1^
*c* = 19.8042 (4) Å	*T* = 100 K
β = 97.552 (1)°	Block, colourless
*V* = 2969.74 (11) Å^3^	0.39 × 0.33 × 0.28 mm
*Z* = 8	

### Data collection

**Table:**

Bruker Kappa APEXII DUO CCD diffractometer	9973 independent reflections
Radiation source: fine-focus sealed tube	8213 reflections with *I* > 2σ(*I*)
Curved graphite crystal monochromator	*R*_int_ = 0.027
Detector resolution: 16 pixels mm^-1^	θ_max_ = 31.7°, θ_min_ = 1.6°
φ scans and ω scans with κ offset	*h* = −28→28
Absorption correction: multi-scan (*SADABS*; Sheldrick, 2008)	*k* = −11→11
*T*_min_ = 0.964, *T*_max_ = 0.974	*l* = −29→29
64574 measured reflections	

### Refinement

**Table:**

Refinement on *F*^2^	Primary atom site location: structure-invariant direct methods
Least-squares matrix: full	Secondary atom site location: difference Fourier map
*R*[*F*^2^ > 2σ(*F*^2^)] = 0.038	Hydrogen site location: difference Fourier map
*wR*(*F*^2^) = 0.106	H atoms treated by a mixture of independent and constrained refinement
*S* = 1.03	*w* = 1/[σ^2^(*F*_o_^2^) + (0.0555*P*)^2^ + 0.8835*P*] where *P* = (*F*_o_^2^ + 2*F*_c_^2^)/3
9973 reflections	(Δ/σ)_max_ = 0.001
419 parameters	Δρ_max_ = 0.49 e Å^−^^3^
0 restraints	Δρ_min_ = −0.25 e Å^−^^3^

### Special details

**Table:**

Geometry. All esds (except the esd in the dihedral angle between two l.s. planes) are estimated using the full covariance matrix. The cell esds are taken into account individually in the estimation of esds in distances, angles and torsion angles; correlations between esds in cell parameters are only used when they are defined by crystal symmetry. An approximate (isotropic) treatment of cell esds is used for estimating esds involving l.s. planes.
Refinement. Refinement of F^2^ against ALL reflections. The weighted R-factor wR and goodness of fit S are based on F^2^, conventional R-factors R are based on F, with F set to zero for negative F^2^. The threshold expression of F^2^ > 2sigma(F^2^) is used only for calculating R-factors(gt) etc. and is not relevant to the choice of reflections for refinement. R-factors based on F^2^ are statistically about twice as large as those based on F, and R- factors based on ALL data will be even larger.

### Fractional atomic coordinates and isotropic or equivalent isotropic displacement parameters (Å^2^)

**Table:**

	*x*	*y*	*z*	*U*_iso_*/*U*_eq_	
O1	0.23337 (4)	0.28795 (10)	0.54568 (4)	0.02361 (15)	
O2	0.24501 (3)	0.03509 (9)	0.75693 (3)	0.01915 (13)	
H2O	0.2117 (9)	−0.025 (2)	0.7803 (8)	0.046 (4)*	
O3	0.45992 (4)	0.18904 (10)	0.25984 (4)	0.02138 (14)	
O4	0.24039 (3)	−0.02793 (9)	0.25464 (3)	0.01929 (13)	
H4O	0.2170 (9)	−0.087 (2)	0.2888 (9)	0.052 (5)*	
N1	0.09028 (4)	0.25022 (10)	0.52885 (4)	0.01688 (14)	
H2N	0.1179 (7)	0.1554 (19)	0.6202 (7)	0.030 (4)*	
N2	0.13341 (4)	0.20263 (11)	0.58605 (4)	0.01772 (15)	
N3	0.20653 (4)	0.08553 (10)	0.69785 (4)	0.01612 (14)	
N4	0.31385 (4)	0.18287 (9)	0.66691 (4)	0.01408 (13)	
N5	0.34090 (4)	0.34130 (10)	0.68560 (4)	0.01503 (14)	
N6	0.47175 (4)	0.22400 (11)	0.40050 (4)	0.01828 (15)	
H7N	0.3769 (8)	0.131 (2)	0.3781 (7)	0.033 (4)*	
N7	0.41368 (4)	0.16597 (11)	0.36033 (4)	0.01910 (15)	
N8	0.30122 (4)	0.02189 (10)	0.29165 (4)	0.01648 (14)	
N9	0.33140 (4)	0.11492 (9)	0.18502 (4)	0.01428 (13)	
N10	0.31523 (4)	0.27505 (9)	0.15885 (4)	0.01431 (13)	
N11	−0.08655 (4)	0.22180 (11)	0.46263 (4)	0.01968 (15)	
N16	0.53482 (4)	0.25441 (12)	0.57576 (4)	0.02314 (17)	
C1	0.00368 (5)	0.38205 (12)	0.41941 (4)	0.01765 (16)	
H1	0.0500	0.4235	0.4260	0.021*	
C2	−0.04025 (5)	0.42371 (13)	0.36098 (5)	0.02003 (17)	
H2	−0.0242	0.4911	0.3262	0.024*	
C3	−0.10829 (5)	0.36503 (14)	0.35412 (5)	0.02119 (18)	
H3	−0.1399	0.3915	0.3147	0.025*	
C4	−0.12863 (5)	0.26698 (14)	0.40633 (5)	0.02197 (18)	
H4	−0.1754	0.2292	0.4020	0.026*	
C5	−0.02103 (4)	0.27821 (12)	0.46846 (4)	0.01566 (15)	
C6	0.02508 (4)	0.22338 (12)	0.53059 (4)	0.01642 (16)	
C7	−0.00595 (5)	0.14381 (14)	0.58873 (5)	0.02139 (18)	
H7A	0.0014	0.2204	0.6284	0.032*	
H7B	0.0163	0.0326	0.6004	0.032*	
H7C	−0.0557	0.1266	0.5754	0.032*	
C8	0.20300 (4)	0.22472 (11)	0.58951 (4)	0.01588 (15)	
C9	0.24134 (4)	0.16000 (11)	0.65548 (4)	0.01431 (15)	
C10	0.40910 (4)	0.31716 (12)	0.69744 (4)	0.01572 (15)	
C11	0.45625 (5)	0.46298 (13)	0.72087 (5)	0.02263 (18)	
H11A	0.4290	0.5586	0.7357	0.034*	
H11B	0.4807	0.5012	0.6833	0.034*	
H11C	0.4900	0.4249	0.7590	0.034*	
C12	0.42576 (4)	0.14436 (12)	0.68606 (4)	0.01676 (16)	
H12	0.4708	0.0952	0.6904	0.020*	
C13	0.36410 (5)	0.06078 (11)	0.66743 (4)	0.01532 (15)	
C14	0.34846 (6)	−0.12230 (12)	0.64969 (5)	0.02392 (19)	
H14A	0.3254	−0.1296	0.6027	0.036*	
H14B	0.3180	−0.1697	0.6807	0.036*	
H14C	0.3917	−0.1884	0.6540	0.036*	
C15	0.58299 (5)	0.37652 (12)	0.48087 (5)	0.01906 (17)	
H15	0.5786	0.3991	0.4334	0.023*	
C16	0.64057 (5)	0.43270 (14)	0.52371 (5)	0.02242 (18)	
H16	0.6768	0.4924	0.5059	0.027*	
C17	0.64462 (5)	0.40039 (15)	0.59324 (5)	0.0246 (2)	
H17	0.6834	0.4378	0.6239	0.030*	
C18	0.59051 (5)	0.31215 (15)	0.61631 (5)	0.0256 (2)	
H18	0.5930	0.2913	0.6638	0.031*	
C19	0.53144 (5)	0.28602 (12)	0.50876 (5)	0.01802 (16)	
C20	0.46937 (5)	0.21770 (12)	0.46535 (5)	0.01815 (16)	
C21	0.41019 (5)	0.14593 (15)	0.49801 (5)	0.02440 (19)	
H21A	0.3683	0.2132	0.4836	0.037*	
H21B	0.4213	0.1520	0.5477	0.037*	
H21C	0.4023	0.0257	0.4841	0.037*	
C22	0.41232 (5)	0.15523 (11)	0.29161 (4)	0.01576 (15)	
C23	0.34393 (4)	0.09303 (11)	0.25621 (4)	0.01461 (15)	
C24	0.30610 (4)	0.25375 (12)	0.09149 (4)	0.01495 (15)	
C25	0.28426 (5)	0.40217 (13)	0.04578 (5)	0.02124 (18)	
H25A	0.2684	0.4966	0.0727	0.032*	
H25B	0.2464	0.3662	0.0111	0.032*	
H25C	0.3236	0.4411	0.0236	0.032*	
C26	0.31770 (5)	0.08076 (12)	0.07453 (4)	0.01728 (16)	
H26	0.3155	0.0333	0.0301	0.021*	
C27	0.33293 (5)	−0.00536 (11)	0.13529 (4)	0.01654 (16)	
C28	0.34777 (6)	−0.18986 (13)	0.15142 (6)	0.0263 (2)	
H28A	0.3918	−0.1995	0.1815	0.039*	
H28B	0.3509	−0.2533	0.1092	0.039*	
H28C	0.3104	−0.2383	0.1742	0.039*	

### Atomic displacement parameters (Å^2^)

**Table:**

	*U*^11^	*U*^22^	*U*^33^	*U*^12^	*U*^13^	*U*^23^
O1	0.0180 (3)	0.0324 (4)	0.0200 (3)	−0.0017 (3)	0.0009 (2)	0.0084 (3)
O2	0.0143 (3)	0.0255 (3)	0.0163 (3)	−0.0025 (2)	−0.0030 (2)	0.0069 (2)
O3	0.0169 (3)	0.0272 (3)	0.0204 (3)	−0.0039 (3)	0.0040 (2)	−0.0007 (3)
O4	0.0160 (3)	0.0253 (3)	0.0161 (3)	−0.0069 (2)	0.0004 (2)	0.0022 (2)
N1	0.0141 (3)	0.0204 (3)	0.0149 (3)	0.0017 (3)	−0.0026 (2)	0.0008 (3)
N2	0.0129 (3)	0.0239 (4)	0.0154 (3)	0.0002 (3)	−0.0019 (3)	0.0040 (3)
N3	0.0140 (3)	0.0177 (3)	0.0155 (3)	−0.0008 (3)	−0.0022 (2)	0.0023 (3)
N4	0.0114 (3)	0.0138 (3)	0.0166 (3)	−0.0002 (2)	0.0003 (2)	0.0001 (3)
N5	0.0129 (3)	0.0145 (3)	0.0173 (3)	−0.0012 (2)	0.0005 (2)	−0.0010 (3)
N6	0.0155 (3)	0.0217 (4)	0.0167 (3)	−0.0014 (3)	−0.0011 (3)	−0.0006 (3)
N7	0.0158 (3)	0.0260 (4)	0.0152 (3)	−0.0046 (3)	0.0007 (3)	−0.0001 (3)
N8	0.0152 (3)	0.0177 (3)	0.0161 (3)	−0.0020 (3)	0.0005 (3)	0.0012 (3)
N9	0.0167 (3)	0.0135 (3)	0.0129 (3)	−0.0001 (2)	0.0025 (2)	0.0001 (2)
N10	0.0146 (3)	0.0148 (3)	0.0135 (3)	0.0014 (2)	0.0016 (2)	0.0007 (2)
N11	0.0137 (3)	0.0266 (4)	0.0181 (3)	−0.0003 (3)	−0.0002 (3)	−0.0005 (3)
N16	0.0203 (4)	0.0316 (4)	0.0172 (3)	0.0015 (3)	0.0009 (3)	−0.0005 (3)
C1	0.0149 (4)	0.0220 (4)	0.0155 (4)	0.0001 (3)	0.0001 (3)	−0.0009 (3)
C2	0.0201 (4)	0.0246 (4)	0.0148 (4)	0.0016 (3)	0.0001 (3)	−0.0002 (3)
C3	0.0176 (4)	0.0294 (5)	0.0152 (4)	0.0043 (3)	−0.0031 (3)	−0.0027 (3)
C4	0.0132 (4)	0.0321 (5)	0.0195 (4)	0.0000 (3)	−0.0020 (3)	−0.0028 (4)
C5	0.0132 (4)	0.0186 (4)	0.0146 (3)	0.0022 (3)	−0.0002 (3)	−0.0022 (3)
C6	0.0146 (4)	0.0194 (4)	0.0147 (4)	0.0015 (3)	−0.0001 (3)	−0.0006 (3)
C7	0.0169 (4)	0.0288 (5)	0.0184 (4)	0.0009 (3)	0.0021 (3)	0.0037 (3)
C8	0.0141 (4)	0.0171 (4)	0.0155 (3)	0.0003 (3)	−0.0014 (3)	0.0007 (3)
C9	0.0118 (3)	0.0148 (3)	0.0155 (3)	−0.0005 (3)	−0.0010 (3)	0.0002 (3)
C10	0.0126 (4)	0.0201 (4)	0.0142 (3)	−0.0009 (3)	0.0009 (3)	0.0011 (3)
C11	0.0165 (4)	0.0241 (4)	0.0263 (4)	−0.0054 (3)	−0.0005 (3)	−0.0009 (4)
C12	0.0130 (4)	0.0218 (4)	0.0155 (4)	0.0033 (3)	0.0019 (3)	0.0017 (3)
C13	0.0161 (4)	0.0164 (4)	0.0135 (3)	0.0030 (3)	0.0021 (3)	0.0006 (3)
C14	0.0264 (5)	0.0166 (4)	0.0287 (5)	0.0028 (3)	0.0034 (4)	−0.0036 (3)
C15	0.0176 (4)	0.0210 (4)	0.0185 (4)	0.0024 (3)	0.0019 (3)	−0.0019 (3)
C16	0.0162 (4)	0.0257 (4)	0.0249 (4)	0.0021 (3)	0.0010 (3)	−0.0042 (4)
C17	0.0165 (4)	0.0332 (5)	0.0229 (4)	0.0050 (4)	−0.0021 (3)	−0.0070 (4)
C18	0.0216 (4)	0.0367 (5)	0.0175 (4)	0.0052 (4)	−0.0005 (3)	−0.0023 (4)
C19	0.0167 (4)	0.0204 (4)	0.0164 (4)	0.0030 (3)	0.0004 (3)	−0.0018 (3)
C20	0.0165 (4)	0.0205 (4)	0.0172 (4)	0.0007 (3)	0.0015 (3)	−0.0002 (3)
C21	0.0223 (4)	0.0314 (5)	0.0197 (4)	−0.0058 (4)	0.0034 (3)	0.0005 (4)
C22	0.0156 (4)	0.0151 (4)	0.0161 (4)	0.0001 (3)	0.0004 (3)	0.0005 (3)
C23	0.0154 (4)	0.0145 (3)	0.0138 (3)	0.0001 (3)	0.0012 (3)	0.0008 (3)
C24	0.0116 (3)	0.0198 (4)	0.0134 (3)	0.0001 (3)	0.0015 (3)	−0.0001 (3)
C25	0.0212 (4)	0.0259 (4)	0.0162 (4)	0.0049 (3)	0.0012 (3)	0.0030 (3)
C26	0.0159 (4)	0.0212 (4)	0.0149 (4)	−0.0013 (3)	0.0027 (3)	−0.0037 (3)
C27	0.0158 (4)	0.0162 (4)	0.0183 (4)	−0.0019 (3)	0.0048 (3)	−0.0031 (3)
C28	0.0349 (5)	0.0151 (4)	0.0311 (5)	−0.0007 (4)	0.0126 (4)	−0.0015 (4)

### Geometric parameters (Å, º)

**Table:**

O1—C8	1.2162 (11)	C7—H7B	0.9800
O2—N3	1.3615 (9)	C7—H7C	0.9800
O2—H2O	0.964 (17)	C8—C9	1.5035 (12)
O3—C22	1.2156 (11)	C10—C12	1.4061 (13)
O4—N8	1.3645 (10)	C10—C11	1.4931 (13)
O4—H4O	0.978 (18)	C11—H11A	0.9800
N1—C6	1.2915 (11)	C11—H11B	0.9800
N1—N2	1.3693 (10)	C11—H11C	0.9800
N2—C8	1.3589 (11)	C12—C13	1.3721 (12)
N2—H2N	0.859 (15)	C12—H12	0.9500
N3—C9	1.2836 (11)	C13—C14	1.4868 (13)
N4—C13	1.3619 (11)	C14—H14A	0.9800
N4—N5	1.3703 (10)	C14—H14B	0.9800
N4—C9	1.4115 (11)	C14—H14C	0.9800
N5—C10	1.3313 (11)	C15—C16	1.3846 (13)
N6—C20	1.2921 (12)	C15—C19	1.3974 (13)
N6—N7	1.3705 (11)	C15—H15	0.9500
N7—C22	1.3603 (11)	C16—C17	1.3916 (14)
N7—H7N	0.882 (15)	C16—H16	0.9500
N8—C23	1.2819 (11)	C17—C18	1.3840 (15)
N9—C27	1.3607 (11)	C17—H17	0.9500
N9—N10	1.3683 (10)	C18—H18	0.9500
N9—C23	1.4090 (11)	C19—C20	1.4862 (13)
N10—C24	1.3327 (11)	C20—C21	1.5010 (13)
N11—C5	1.3396 (11)	C21—H21A	0.9800
N11—C4	1.3408 (12)	C21—H21B	0.9800
N16—C18	1.3391 (13)	C21—H21C	0.9800
N16—C19	1.3425 (12)	C22—C23	1.5010 (12)
C1—C2	1.3841 (12)	C24—C26	1.4105 (13)
C1—C5	1.3959 (12)	C24—C25	1.4927 (13)
C1—H1	0.9500	C25—H25A	0.9800
C2—C3	1.3909 (14)	C25—H25B	0.9800
C2—H2	0.9500	C25—H25C	0.9800
C3—C4	1.3834 (14)	C26—C27	1.3747 (13)
C3—H3	0.9500	C26—H26	0.9500
C4—H4	0.9500	C27—C28	1.4884 (13)
C5—C6	1.4872 (12)	C28—H28A	0.9800
C6—C7	1.5019 (13)	C28—H28B	0.9800
C7—H7A	0.9800	C28—H28C	0.9800
			
N3—O2—H2O	102.8 (10)	C13—C12—H12	126.8
N8—O4—H4O	102.3 (10)	C10—C12—H12	126.8
C6—N1—N2	115.63 (8)	N4—C13—C12	105.97 (8)
C8—N2—N1	120.46 (8)	N4—C13—C14	122.66 (8)
C8—N2—H2N	117.7 (10)	C12—C13—C14	131.37 (8)
N1—N2—H2N	121.8 (10)	C13—C14—H14A	109.5
C9—N3—O2	114.41 (7)	C13—C14—H14B	109.5
C13—N4—N5	111.90 (7)	H14A—C14—H14B	109.5
C13—N4—C9	128.04 (8)	C13—C14—H14C	109.5
N5—N4—C9	119.83 (7)	H14A—C14—H14C	109.5
C10—N5—N4	105.09 (7)	H14B—C14—H14C	109.5
C20—N6—N7	115.45 (8)	C16—C15—C19	118.83 (9)
C22—N7—N6	120.46 (8)	C16—C15—H15	120.6
C22—N7—H7N	118.1 (10)	C19—C15—H15	120.6
N6—N7—H7N	121.4 (10)	C15—C16—C17	119.05 (9)
C23—N8—O4	114.08 (7)	C15—C16—H16	120.5
C27—N9—N10	112.07 (7)	C17—C16—H16	120.5
C27—N9—C23	128.60 (8)	C18—C17—C16	117.99 (9)
N10—N9—C23	119.32 (7)	C18—C17—H17	121.0
C24—N10—N9	105.08 (7)	C16—C17—H17	121.0
C5—N11—C4	117.20 (8)	N16—C18—C17	124.04 (9)
C18—N16—C19	117.51 (9)	N16—C18—H18	118.0
C2—C1—C5	119.02 (8)	C17—C18—H18	118.0
C2—C1—H1	120.5	N16—C19—C15	122.57 (9)
C5—C1—H1	120.5	N16—C19—C20	115.91 (8)
C1—C2—C3	118.76 (9)	C15—C19—C20	121.53 (8)
C1—C2—H2	120.6	N6—C20—C19	115.39 (8)
C3—C2—H2	120.6	N6—C20—C21	124.91 (8)
C4—C3—C2	118.04 (8)	C19—C20—C21	119.70 (8)
C4—C3—H3	121.0	C20—C21—H21A	109.5
C2—C3—H3	121.0	C20—C21—H21B	109.5
N11—C4—C3	124.19 (9)	H21A—C21—H21B	109.5
N11—C4—H4	117.9	C20—C21—H21C	109.5
C3—C4—H4	117.9	H21A—C21—H21C	109.5
N11—C5—C1	122.73 (8)	H21B—C21—H21C	109.5
N11—C5—C6	116.25 (8)	O3—C22—N7	126.06 (8)
C1—C5—C6	121.02 (8)	O3—C22—C23	121.19 (8)
N1—C6—C5	114.87 (8)	N7—C22—C23	112.74 (7)
N1—C6—C7	125.65 (8)	N8—C23—N9	124.08 (8)
C5—C6—C7	119.48 (8)	N8—C23—C22	118.86 (8)
C6—C7—H7A	109.5	N9—C23—C22	117.06 (7)
C6—C7—H7B	109.5	N10—C24—C26	110.62 (8)
H7A—C7—H7B	109.5	N10—C24—C25	120.07 (8)
C6—C7—H7C	109.5	C26—C24—C25	129.28 (8)
H7A—C7—H7C	109.5	C24—C25—H25A	109.5
H7B—C7—H7C	109.5	C24—C25—H25B	109.5
O1—C8—N2	126.18 (8)	H25A—C25—H25B	109.5
O1—C8—C9	121.51 (8)	C24—C25—H25C	109.5
N2—C8—C9	112.31 (7)	H25A—C25—H25C	109.5
N3—C9—N4	123.70 (8)	H25B—C25—H25C	109.5
N3—C9—C8	118.31 (8)	C27—C26—C24	106.14 (8)
N4—C9—C8	117.99 (7)	C27—C26—H26	126.9
N5—C10—C12	110.61 (8)	C24—C26—H26	126.9
N5—C10—C11	120.42 (8)	N9—C27—C26	106.07 (8)
C12—C10—C11	128.96 (8)	N9—C27—C28	121.87 (8)
C10—C11—H11A	109.5	C26—C27—C28	132.05 (9)
C10—C11—H11B	109.5	C27—C28—H28A	109.5
H11A—C11—H11B	109.5	C27—C28—H28B	109.5
C10—C11—H11C	109.5	H28A—C28—H28B	109.5
H11A—C11—H11C	109.5	C27—C28—H28C	109.5
H11B—C11—H11C	109.5	H28A—C28—H28C	109.5
C13—C12—C10	106.42 (8)	H28B—C28—H28C	109.5

### Hydrogen-bond geometry (Å, º)

**Table:**

*D*—H···*A*	*D*—H	H···*A*	*D*···*A*	*D*—H···*A*
O2—H2*O*···N5^i^	0.964 (17)	1.664 (17)	2.6193 (10)	170.1 (16)
O4—H4*O*···N10^ii^	0.978 (18)	1.670 (18)	2.6341 (10)	167.8 (17)

Symmetry codes: (i) −*x*+1/2, *y*−1/2, −*z*+3/2; (ii) −*x*+1/2, *y*−1/2, −*z*+1/2.
